# Exploring the Prakriti (Body Constitution) of Stroke Patients: A Scoping Review From an Ayurvedic Perspective

**DOI:** 10.7759/cureus.102368

**Published:** 2026-01-27

**Authors:** Shravanthi S, Sanketh V Sharma, Amritha Sindhu, Arun Bhanu K, Harikrishnan M, Subrahmanya Kumar Kukkupuni, Chethala N Vishnuprasad, Komal Prasad Chandrachari, Yogesh Shouche, Lavanya Garady, Prasan Shankar

**Affiliations:** 1 Ayurveda Biology and Holistic Nutrition, The University of Trans-Disciplinary Health Sciences and Technology, Bengaluru, IND; 2 School of Humanities, National Institute of Advanced Studies (NIAS), Bengaluru, IND; 3 Department of Neurosurgery, Narayana Institute of Neurosciences/Mazumdar Shaw Medical Center, Bengaluru, IND; 4 Department of Microbiology, Scientific Knowledge for Ageing and Neurological Ailments (SKAN) Research Trust, Bengaluru, IND; 5 Department of Public Health Sciences, Scientific Knowledge for Ageing and Neurological Ailments (SKAN) Research Trust, Bengaluru, IND; 6 Rasayana Tantra Unit, I-AIM Healthcare Center, The University of Trans-Disciplinary Health Sciences and Technology, Bengaluru, IND

**Keywords:** ayurveda, body constitution, hemiplegia, prakriti, risk prediction, stroke

## Abstract

The ever-rising burden of stroke is a major problem for developing nations with inadequate resources, such as India. Current strategies largely focus on the prevention and control of non-communicable diseases (NCDs); however, there is a growing need to shift toward predictive approaches that enable timely lifestyle modification and risk reduction. Ayurveda’s concept of *Prakriti*, the distinctive body constitution or psychosomatic temperament of an individual, offers a novel framework for understanding disease predisposition.

This scoping review aimed to provide a comprehensive overview of studies that have examined the classification of *Prakriti* among stroke patients. Online search engines were used to conduct a literature search using both Sanskrit and English keywords, employing Boolean operators to combine relevant terms. Full-text articles on stroke, hemiplegia, or *Pakshaghata* were retrieved, screened, and included based on the presence of *Prakriti* analysis.

A total of 27 studies met the inclusion criteria, comprising 13 randomized controlled or quasi-experimental studies, 12 case studies or case reports, and 2 cross-sectional studies. Across these studies, *Vata*-dominant *Prakriti* (*Vataja*, *Vata-Pittaja*, or *Vata-Kaphaja*) was reported in 24 out of 27 studies, either independently or in combination with another Dosha. Only three studies reported a predominance of other constitutions.

This scoping review highlights a notable gap in existing stroke research, wherein *Prakriti *assessment is frequently overlooked. Integrating *Prakriti*-based profiling into predictive medicine may enhance individualized stroke risk assessment and contribute to more personalized preventive strategies.

## Introduction and background

Stroke, also known as cerebrovascular accident (CVA), occurs due to the interruption of blood supply to the brain and is broadly classified into ischemic and hemorrhagic types [1]. Stroke remains a major global public health challenge, ranking as the second leading cause of death and third leading cause of disability worldwide. According to the Global Burden of Disease (GBD) Study 2019, stroke accounted for approximately 6.55 million deaths and 143 million disability-adjusted life years (DALYs) globally, with 12.2 million incident cases and 101 million prevalent cases [2]. The global burden of stroke has increased substantially over the past three decades, with stroke-related deaths and DALYs rising by 43% and 32%, respectively, between 1990 and 2019 [2]. Notably, 86%-89% of stroke-related deaths and DALYs occur in low- and middle-income countries (LMICs), where stroke mortality is 3.6 times higher than in high-income countries [3].

In India, the burden of stroke has shown a steady upward trend, with stroke ranking as the third leading cause of death and sixth leading cause of disability. In 2021, stroke accounted for 6.58% of total deaths and 3.67% of total DALYs, with crude incidence rates ranging from 108 to 172 per 100,000 population per year [4-6]. Stroke-related deaths in India increased by approximately 25% between 1990 and 2021 [7].

Importantly, stroke is no longer confined to older populations. Globally, individuals below 70 years of age accounted for nearly 63% of stroke cases, and it is estimated that one in four individuals will experience a stroke during their lifetime [8]. In India, strokes occur at a younger mean age, nearly 15 years earlier than in high-income countries, with 10%-15% of all strokes occurring among younger adults, contributing to a substantial socioeconomic burden [9-12].

In the GBD Study, 19 risk factors were shown to contribute to stroke-related DALYs. The leading external predisposing factors associated with stroke-related death and disability include high systolic blood pressure, elevated body mass index, high fasting plasma glucose, ambient particulate matter pollution, and tobacco use [2]. Additional contributors include high total cholesterol levels, reduced glomerular filtration rate, unhealthy dietary patterns, and low physical activity [8]. These factors largely represent modifiable behavioral, metabolic, and environmental exposures that influence stroke risk across populations. Despite advancing healthcare with an increasing Universal Health Coverage (UHC) Service Coverage Index from 29.66 in 2000 to 63.66 in 2021, a steadily rising trend of stroke burden across different geographical regions in India has highlighted the urgent need for enhanced stroke prediction and prevention strategies [7]. Given the multifactorial nature of stroke and its substantial burden of premature morbidity and disability, early identification of individuals at increased risk is essential. Conventional preventive strategies often focus on risk factor modification after clinical manifestation, potentially overlooking individuals with underlying biological susceptibility. This highlights the need for predictive approaches that can identify risk at an earlier stage, enabling timely preventive interventions and personalized lifestyle modification.

Predictive medicine is defined as “a branch of medicine that aims to identify patients at risk of developing a disease, thereby enabling either prevention or early treatment of that disease,” and has gained increasing relevance in the context of chronic non-communicable diseases (NCDs) such as stroke [13]. Ayurveda, the traditional Indian System of Medicine developed over 5000 years ago, has predictive, preventive, and personalized medicine ingrained in its roots [14]. It presents the concept of Prakriti, a foundational principle of Ayurveda that describes the distinctive body constitution or psychosomatic temperament of an individual.

Ayurvedic principles state that every individual is said to have three *Dosha*, namely, "*Vata Dosha*," "*Pitta Dosha*," and "*Kapha Dosha*." The predominance of one or more of these three *Dosha* (biofunctional principles) determines an individual’s *Prakriti *[15,16]. Hence, *Prakriti *represents the biological constitution of an individual with unique physical, physiological, and psychological characteristics. It is determined at the time of conception and remains constant throughout a person’s lifetime [14].

Ayurvedic classical texts describe two principal types of *Prakriti-Sharirika* (*Deha*) *Prakriti*, which pertains to the physical constitution, and *Manasika Prakriti*, which relates to psychological attributes. The present review focuses exclusively on *Sharirika* (*Deha*) *Prakriti*, which is classified into seven types based on the dominance of the three *Dosha* (biofunctional principles): (1)* Vataja Prakriti*, (2) *Pittaja Prakriti*, (3) *Kaphaja Prakriti*, (4) *Vata*-*Pittaja Prakriti*, (5) *Vata*-*Kaphaja* *Prakriti*, (6) *Pitta*-*Kaphaja* *Prakriti*, and (7) *Sama *(*Tridoshaja*) *Prakriti  *[16]. 

Individuals belonging to each type of *Prakriti *are susceptible to certain diseases arising from the vitiation of their respective *Dosha* (biofunctional principles) without any contribution from other *Dosha*, which are referred to as "*Nanatmaja Vikara*"* *[14]. For instance, *Vata Prakriti *individuals are prone to 80 diseases born out of the *Vata Dosha *vitiation alone, referred to as "*Vataja Nanatmaja Vikara*." Hemiparesis and hemiplegia, common clinical manifestations of stroke, correlate with the disease *Pakshaghata*/*Pakshavadha* described in Ayurveda and are listed among the 80 *Vataja Nanatmaja Vikara* [17]. According to the proponents of Ayurveda, its pathogenesis involves *Vata Dosha *getting lodged in one half of the body, resulting in weakness and a loss of motor and sensory function in that half [18].

*Prakriti*'s role in Ayurgenomics, a tailored methodology in P4 medicine (predictive, preventive, personalized, and participatory), has been gaining traction recently [16,19]. The concept of *Prakriti *parallels the principles of epigenetics, as both frameworks integrate genotypic predispositions with phenotypic expression to explain inter-individual variability in health and disease. Despite this conceptual alignment, limited research has explored *Prakriti *in the context of stroke, one of the leading causes of disability and mortality worldwide. Understanding the distribution of *Prakriti* among stroke patients could provide valuable insights into individual susceptibility, thereby informing predictive and preventive healthcare strategies. Hence, this scoping review seeks to provide a comprehensive overview of existing studies that have examined the classification of *Prakriti *among stroke patients, contributing to the growing evidence base that supports Ayurveda’s role in predictive, personalized, and integrative medicine.

## Review

Methods

The PRISMA Extension for Scoping Reviews (PRISMA-ScR) checklist was utilized to guide the reporting of this review [20].

Eligibility Criteria

Studies were considered eligible for inclusion if they focused on patients with stroke, hemiplegia, or *Pakshaghata *and reported an analysis of the *Prakriti *of the participants. Both interventional and observational designs were considered, including randomized controlled trials (RCTs), quasi-experimental studies, cross-sectional studies, and case studies. Studies without *Prakriti *assessment, as well as review articles and abstracts without full-text availability, were excluded.

Information Sources

A comprehensive search was conducted across multiple open-access databases and search engines to ensure wide coverage of published literature. The following sources were systematically explored: PubMed, Google, Google Scholar, and the AYUSH Research Portal.

Search Strategy

A comprehensive literature search was conducted to identify studies examining the association between *Prakriti* (Ayurvedic constitutional types) and stroke. The search was performed across multiple electronic databases, including PubMed, Google Scholar, and AYUSH Research Portal, from database inception to 2025.

The core search strategy included terms related to Ayurveda and constitutional phenotypes (“*Prakriti*,” “*Deha Prakriti*”), combined using Boolean operators with cardiovascular outcomes (“stroke”). 

The search was limited to articles published in English. Reference lists of included articles and relevant reviews were also manually screened to identify additional eligible studies.

Data Selection and Extraction 

All retrieved records were initially screened for eligibility based on titles and abstracts. Potentially relevant articles were then evaluated in full text against the inclusion and exclusion criteria. For each included study, a structured data extraction form was prepared to capture relevant details such as study design, year of publication, sample size, distribution of *Prakriti *among participants, etc. The extracted data were systematically tabulated (Table 1).

**Table 1:** Data from included articles

Search engine	Author (year)	Study design	Diagnosis	Sample size and population	*Prakriti* analysis
AYUSH Research Portal	Naphade et al. (2011) [21]	Single-blind study (RCT)	Thromboembolic infarction	30 patients aged 30-70 years	14 (46.66%), *Vataja Prakriti*
					10 (33.33%), *Vata-Pittaja Prakriti*
AYUSH Research Portal	Desai et al. (1988) [22]	Comparative study (RCT)	Based on clinical symptoms	12 patients	8 (66.66%), *Dvandvaja Prakriti* with the dominance of *Vata-Kaphaja Prakriti*
AYUSH Research Portal	Masatkar (2018) [23]	Open randomized study (RCT)	Ischemic stroke	20 patients aged 40-70 years	10 (50%), *Vata-Kaphaja Prakriti*
					7 (35%), *Vata-Pittaja Prakriti*
					3 (15%), *Pitta-Kaphaja Prakriti*
AYUSH Research Portal	Rajalaxmi et al. (2014) [24]	RCT	Ischemic stroke	50 patients aged 16-70 years	31 (62%), *Vata-Pittaja Prakriti*
AYUSH Research Portal	Pillai et al. (1980) [25]	RCT	Based on clinical symptoms	112 patients aged more than 10 years	51 (45.53%), *Vata-Pittaja Prakriti*
					46 (41.07%), *Vata-Kaphaja Prakriti*
					15 (13.40%), *Pitta-Kaphaja Prakriti*
AYUSH Research Portal	Namboodiri et al. (1980) [26]	RCT	Internal capsule artery thrombosis, 94	109 patients aged more than 10 years	77 (70.64%), *Vata-Kaphaja Prakriti*
			Internal capsule embolism, 4		30 (27.52%), *Vata-Pittaja Prakriti*
			Intracranial hemorrhage, 7		2 (1.84%), *Pitta-Kaphaja Prakriti*
			Others, 4		
AYUSH Research Portal	Dhiman JK et al. (2018) [27]	Case report	Ischemic stroke	54-year-old male	Vata-Pittaja Prakriti
Google Scholar	Rao et al. (2021) [28]	Case report	Ischemic stroke	60-year-old male	Kapha-Vataja Prakriti
Google Scholar	Shukla et al. (2024) [29]	Case report	H/O CVA/stroke	60-year-old male	Vata-Kaphaja Prakriti
Google Scholar	Sindhu et al. (2024) [30]	Case report	H/O ischemic stroke	50-year-old male	Vata-Kaphaja Prakriti
Google Scholar	Febin et al. (2019) [31]	Case report	H/O stroke	68-year-old male	Pitta-Kaphaja Prakriti
Google Scholar	Acharya et al. (2022) [32]	RCT	Vascular stroke	30 patients	13 (43.33%), *Vata-Kaphaja Prakriti*
					11 (36.67%), *Vata-Pittaja Prakriti*
Google Scholar	Kaushish et al. (2023) [33]	RCT	Ischemic stroke	20 patients aged 18-70 years	10 (50%), *Vata-Pittaja Prakriti*
					7 (36%), *Pitta-Kaphaja Prakriti*
					3 (14%), *Vata-Kaphaja Prakriti*
Google Scholar	Kambale et al. (2016) [34]	Cross-sectional study	Ischemic and hemorrhagic stroke	60 patients	27 (45%), *Pitta-Vataja Prakriti*
					19 (31%), *Vata-Kaphaja Prakriti*
					10 (16 %), *Kapha-Vataja Prakriti*
					4 (6%), *Kapha-Pittaja Prakriti*
Google Scholar	Sukanya et al. (2024) [35]	Cross-sectional study	Ischemic and hemorrhagic stroke	96 patients aged 40-70 years	51 (53.1%), *Kapha*-predominant *Prakriti*
					26 (27.1%), *Vata*-predominant *Prakriti*
					19 (19.8%), *Pitta*-predominant *Prakriti*
Google Scholar	Mural et al. (2024) [36]	Case study	Ischemic stroke	48-year-old female	Kapha-Vataja Prakriti
Google	Krishna et al. (2022) [37]	Case study	Ischemic stroke	53-year-old male	Vata-Pittaja Prakriti
Google	Roopa et al. (2022) [38]	Case study	Infarct and intraventricular hemorrhage	61-year-old male	Vata-Kaphaja Prakriti
Google	Menon et al. (2022) [39]	Case report	CVA of thrombotic origin	52-year-old female	Vata-Pittaja Prakriti
Google	Jaju et al. (2022) [40]	Case report	Ischemic stroke	68-year-old female	Vata-Kaphaja Prakriti
Google	Bhagyashree et al. (2023) [41]	Case report	Ischemic stroke	52-year-old male	Vata-Kaphaja Prakriti
Google	Rohidas et al. (2022) [42]	Case report	Infarct	36-year-old male	Vata-Pittaja Prakriti
Google	Singh et al. (2020) [43]	RCT	Based on clinical symptoms	30 patients aged 30-70 years	15 (50%), *Vata-Kaphaja Prakriti*
					9 (30%), *Vata-Pittaja Prakriti*
					6 (20%), *Pitta-Kaphaja Prakriti*
Google	Patil (2017) [44]	RCT	Based on clinical symptoms	60 patients aged 30-70 years	53 (88.3%), *Vata-Pittaja Prakriti*
					6 (10%), *Pitta-Kaphaja Prakriti*
Google	Sharma et al. (2013) [45]	RCT	Based on clinical symptoms	60 patients aged 30-70 years	26 (43.42%), *Pitta-Kaphaja Prakriti*
Google	Chauhan et al. (2018) [46]	Quasi-experimental	CVA of thrombotic origin	26 patients aged 30-75 years	11 (42.3%), *Vata-Pittaja Prakriti*
PubMed	Sankaran et al. (2019) [47]	RCT	Ischemic stroke	25 cases	10 (40%), *Vata-*predominant* Prakriti*
					8 (32%), *Pitta*-predominant* Prakriti*
					7 (28%), *Kapha*-predominant* Prakriti*

Data Analysis

Descriptive statistical methods, including frequency and percentage, were employed to analyze the distribution of *Prakriti *types across the studies. The findings were then synthesized to provide a comprehensive overview of *Prakriti *distribution among stroke patients.

Results

In Ayurveda, stroke (*Pakshaghata*) is primarily caused by vitiation of *Vata Dosha*, sometimes in combination with *Pitta* or *Kapha*. It is classified as *Kevala Vataja Pakshaghata*, *Pittanubandhi Pakshaghata*, and *Kaphanubandhi Pakshaghata*.

For ease of comprehension and accurate interpretation of Ayurvedic terminology presented in this review, readers are encouraged to refer to the Glossary, which provides standardized English translations and brief descriptions of key Sanskrit terms (Table 2).

**Table 2:** Glossary of Sanskrit terms

Sanskrit terminology	Brief description
*Dosha* [48]	The vital governing principles regulating physiological and psychological functions in the body. Their balance ensures health, while an imbalance leads to disease.
*Vata Dosha *[48]	One of the three regulatory functional factors (*Dosha*). The biofunctional principle governing all sensory perceptions, motor activities, and higher mental activities.
*Pitta Dosha *[48]	One of the three regulatory functional factors (*Dosha*). The biofunctional principle governing all digestive and metabolic activities.
*Kapha Dosha *[48]	One of the three regulatory functional factors (*Dosha*). The biofunctional principle governing structural integrity, stability, cohesion, unctuousness, and immunity.
*Prakriti *[49]	*Prakriti*, derived from the Sanskrit word meaning "nature" or "constitution," represents an individual's natural state, determined by their genetic makeup.
*Vataja Prakriti *[49]	The body constitution resulting from dominance of *Vata Dosha*.
*Pittaja Prakriti *[49]	The body constitution resulting from dominance of *Pitta Dosha*.
*Kaphaja Prakriti *[49]	The body constitution resulting from dominance of *Kapha Dosha*.
*Vata-Pittaja Prakriti *[49]	The body constitution resulting from dominance of *Vata *and *Pitta Dosha*.
*Vata-Kaphaja Prakriti *[49]	The body constitution resulting from dominance of *Vata *and *Kapha Dosha*.
*Pitta-Kaphaja Prakriti *[49]	The body constitution resulting from dominance of *Pitta *and *Kapha Dosha*.
*Sama Prakriti *[49]	The body constitution resulting from an equal proportion of *Vata*, *Pitta*,* *and *Kapha Dosha*.
*Pakshaghata *[50]	Hemiplegia/stroke resulting in weakness and a loss of motor and sensory function in one-half of the body.
*Kevala Vataja Pakshaghata *[51]	Hemiplegia caused by the vitiation of *Vata Dosha *alone.
*Pittanubandhi Pakshaghata *[51]	Hemiplegia caused by the vitiation of *Vata *and *Pitta Dosha*.
*Kaphanubandhi Pakshaghata *[51]	Hemiplegia caused by the vitiation of *Vata *and *Kapha Dosha*.

Study Selection

**Figure 1:**
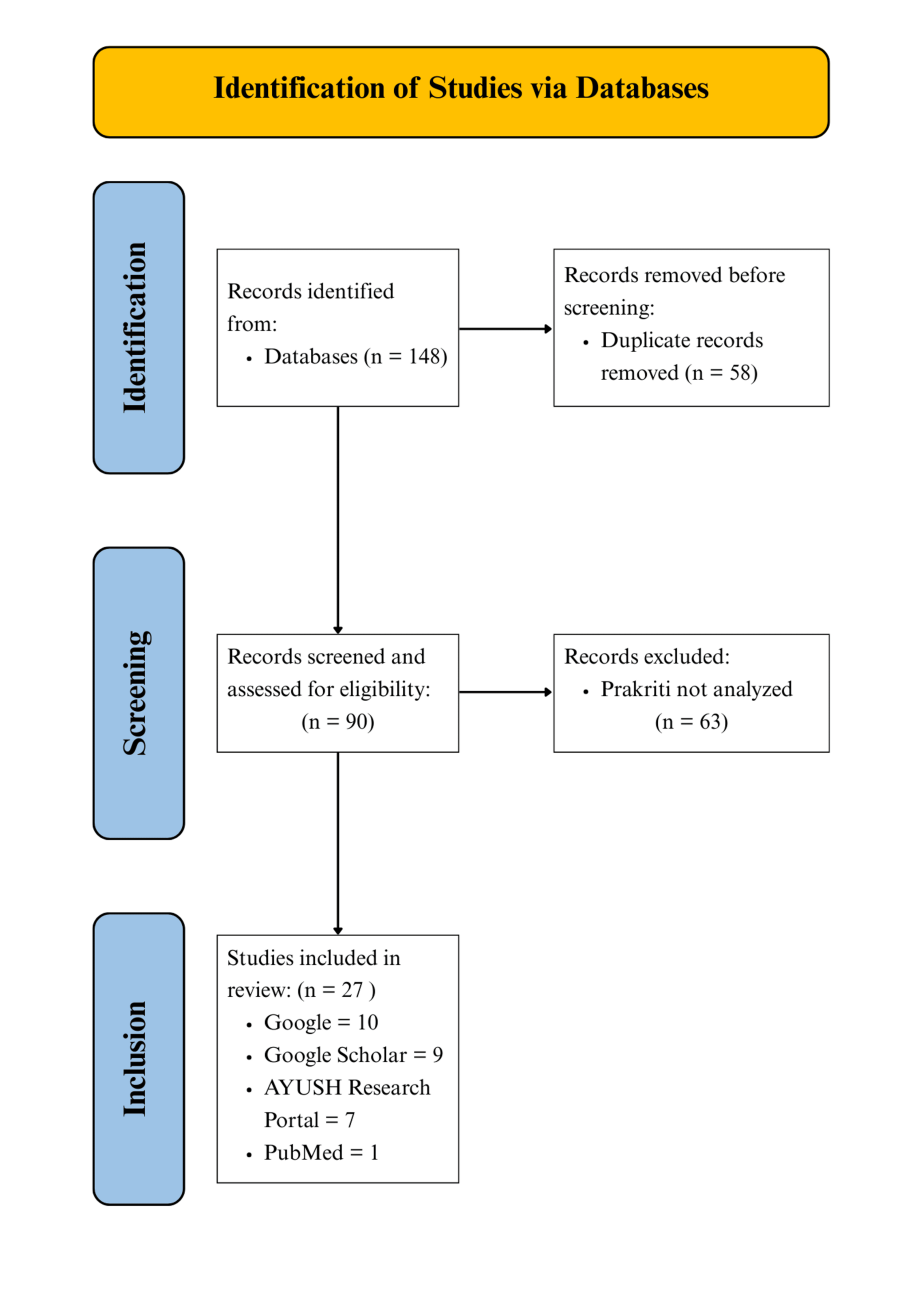
PRISMA-ScR flow diagram of the literature search PRISMA-ScR: Preferred Reporting Items for Systematic reviews and Meta-Analyses extension for Scoping Reviews.

A total of 148 articles were retrieved from various databases, out of which 58 duplicates were removed. The remaining 90 full-text articles were assessed for *Prakriti *analysis in stroke patients. Of these, 63 were excluded as they did not report *Prakriti *assessment. Finally, 27 articles were included in the review (Figure 1).

The characteristics of the selected studies are provided in Table 3.

**Table 3:** Characteristics of the selected articles Source: [22-48].

Characteristics		Frequency (n)	Percentage (%)
Study design	RCT and quasi-experimental study	13	48.2
	Case studies and reports	12	44.4
	Observational cross-sectional studies	2	7.4
Year of publication	1980-1990	3	11.1
	1991-2000	0	0
	2001-2010	0	0
	2011-2020	11	40.7
	2021-2025	13	48.2
Search engine	Google	10	37.1
	Google Scholar	9	33.3
	AYUSH Research Portal	7	25.9
	PubMed	1	3.7

Prakriti Distribution among Stroke Patients

For the purpose of uniformity in data synthesis, similar *Prakriti *categories reported under varying nomenclature were grouped together. Specifically, *Kapha*-*Vataja *and *Vata*-*Kaphaja *were combined as *Vata*-*Kaphaja Prakriti*, while *Pitta*-*Vataja *and *Vata*-*Pittaja *were grouped as *Vata*-*Pittaja Prakriti*, since both pairs represent the same dual *Dosha *dominance with *Vata *as the primary component. This standardization ensured consistency and comparability in the interpretation of *Prakriti *distribution across studies.

The distribution of *Prakriti *types among stroke patients varied across study designs. In the 13 RCTs and quasi-experimental studies reviewed, *Vata*-*Kaphaja *and *Vata*-*Pittaja Prakriti *were reported in five studies each, while *Vataja Prakriti *was reported in two. In seven case studies and reports, *Vata*-*Kaphaja Prakriti *was observed in patients, followed by *Vata*-*Pittaja Prakriti *in four. Among observational studies, one reported *Vata*-*Kaphaja Prakriti *in 47% of patients, while another found *Kaphaja Prakriti *in 53.1% of cases (Table 4).

**Table 4:** Distribution of Prakriti among stroke patients according to the study design Source: [22-48].

Study design	Predominant *Prakriti *type	Frequency (n)	Percentage (%)
RCT and quasi-experimental study (13)	*Vata*-*Kaphaja*	5	38.5
	*Vata*-*Pittaja*	5	38.5
	Vataja	2	15.4
	*Pitta*-*Kaphaja*	1	7.7
Case studies and reports (12)	*Vata*-*Kaphaja*	7	58.4
	*Vata*-*Pittaja*	4	33.3
	*Pitta*-*Kaphaja*	1	8.3
Observational cross-sectional studies (2)	Vata-Kaphaja	1	50
	Kaphaja	1	50

An overall analysis of the included studies revealed that *Vata*-*Kaphaja* *Prakriti *was the most frequently reported type among stroke patients (48.2%), followed by *Vata*-*Pittaja Prakriti *(33.3%) (Table 5).

**Table 5:** Overall distribution of Prakriti among stroke patients in the included studies Source: [22-48].

Predominant *Prakriti *type	Frequency (n)	Percentage (%)
*Vata*-*Kaphaja*	13	48.2
*Vata*-*Pittaja*	9	33.3
Vataja	2	7.4
*Pitta*-*Kaphaja*	2	7.4
Kaphaja	1	3.7

Individuals with *Vata*-*Kaphaja Prakriti *were found to be equally affected by *Kevala Vataja Pakshaghata* (stroke arising solely from *Vata *vitiation) and *Kaphanubandhi Pakshaghata *(stroke arising from both *Vata *and *Kapha Dosha *vitiation). Those with *Vata*-*Pittaja Prakriti *were predominantly afflicted by *Kevala Vataja* *Pakshaghata* (stroke arising solely from *Vata *vitiation), with a smaller proportion experiencing *Kaphanubandhi* and *Pittanubandhi Pakshaghata *(stroke involving the vitiation of *Vata *with *Kapha *or *Pitta*, respectively). Individuals of *Vataja Prakriti *exclusively presented with *Kevala* *Vataja* *Pakshaghata *(stroke arising solely from *Vata *vitiation). Participants with *Pitta*-*Kaphaja Prakriti *showed an equal distribution between *Pittanubandhi *and *Kaphanubandhi* *Pakshaghata *(stroke involving the vitiation of *Vata *with *Pitta *or *Kapha*, respectively). Interestingly, one study reporting a predominance of *Kaphaja Prakriti *individuals observed only *Kevala Vataja Pakshaghata *(stroke arising solely from *Vata *vitiation) cases. However, one study did not specify the subtype of *Pakshaghata *among its participants (Table 6).

**Table 6:** Distribution of Prakriti according to Pakshaghata (stroke) types Source: [22-48].

Predominant *Prakriti *type	*Pakshaghata *(stroke) types		
	*Kevala Vataja* (stroke due to *Vata Dosha* vitiation alone)*, *n (%)	*Kaphanubandhi *(stroke due to *Vata *and *Kapha Dosha* vitiation)*, *n (%)	*Pittanubandhi *(stroke due to *Vata *and *Pitta Dosha* vitiation)*, *n (%)
Vata-Kaphaja	6 (50)	6 (50)	0 (0)
Vata-Pittaja	6 (66.7)	2 (22.2)	1 (11.1)
Vataja	2 (100)	0 (0)	0 (0)
Pitta-Kaphaja	0	1 (50)	1 (50)
Kaphaja	1 (100)	0 (0)	0 (0)

Discussion

The present review sought to examine the distribution of *Prakriti* among stroke patients and identified a consistent trend. In most included studies, patients exhibited a predominance of *Vata Dosha*, either independently or in combination with other *Dosha*. This recurring pattern aligns with the Ayurvedic understanding that derangement of *Vata* is the principal factor in the pathogenesis of *Pakshaghata* (hemiplegia/stroke).

Variations in *Prakriti* distribution across studies may be influenced by several factors. Dual-*dosha* categories such as *Vata-Pittaja* or *Vata-Kaphaja* reflect differences in primary and secondary *Dosha* dominance, which could result in variability depending on how authors classified *Prakriti*. Other contributors include population heterogeneity, stroke subtype differences (ischemic vs hemorrhagic), age, gender, and regional or ethnic factors.

Globally, stroke remains a major public health challenge, with more than 80% of the projected 15 million new cases by 2050 expected to occur in low- and middle-income nations [52]. Developing countries such as India face a disproportionate burden due to limited healthcare infrastructure, high out-of-pocket expenditure, and restricted access to rehabilitation services, resulting in high stroke-related morbidity and mortality [18,53]. While public health efforts emphasize prevention, a shift toward prediction and early risk stratification is needed to facilitate lifestyle modifications and reduce disease incidence.

Current predictive tools such as the Framingham Stroke Risk Score are widely used, but they have limitations in the Indian context, as they do not incorporate ethnic, lifestyle, and constitution-specific factors [54]. Ayurveda provides an indigenous and time-tested approach through the concept of *Prakriti*, which classifies individuals based on their unique constitutional makeup derived from the three *Dosha* (biofunctional principles). Unlike conventional risk tools that rely primarily on measurable biomedical parameters, *Prakriti* assessment is holistic, integrating physical attributes (such as body frame and musculature), physiological tendencies (such as metabolism and digestion), psychological inclinations, and even innate disease susceptibility. Because the same *Dosha* (biofunctional principles) that define *Prakriti* are also responsible for disease causation, *Prakriti* evaluation offers a unique pathway for early identification of at-risk individuals, prediction of disease patterns, preventive strategies, and personalized therapeutic interventions [14,20].

The findings of this review support this premise: individuals with *Vata*-dominant *Prakriti*, particularly *Vataja*, *Vata-Pittaja*, and *Vata-Kaphaja* types, appear more vulnerable to stroke. This potential association emphasizes the relevance of *Prakriti*-based profiling as a potential predictive marker in stroke research and preventive medicine. However, a key research gap persists. Although *Prakriti* analysis shows clinical relevance, its use in stroke-related research remains limited by the absence of standardized, validated, and reproducible diagnostic methods. Current approaches vary widely across studies and often depend on subjective physician assessment, which limits comparability and reliability. Addressing this diagnostic heterogeneity through standardized *Prakriti* assessment tools is essential for meaningful integration into stroke research and personalized risk stratification. Beyond its potential association with stroke risk, *Prakriti *analysis may also help in differentiating stroke subtypes and tailoring therapeutic strategies. 

Ayurvedic texts describe individuals with *Vata*-dominant *Prakriti*, particularly *Vataja*, *Vata-Pittaja*, and *Vata-Kaphaja* types, may be at higher risk of developing stroke if they do not adhere to a healthy lifestyle, including a balanced diet, regular physical activity, and stress management. Classical Ayurvedic texts describe distinct clinical patterns of *Pakshaghata* (hemiplegia) depending on the *Dosha *predominance: *Vataja *cases often manifest with sudden onset, dryness, emaciation, and marked motor deficits; *Vata-Pittaja* types may exhibit burning sensations, irritability, or inflammatory features; whereas *Vata-Kaphaja* strokes tend to present with heaviness, sluggish recovery, and excess secretions. This nuanced differentiation aligns with the heterogeneity observed in modern stroke pathology, where ischemic versus hemorrhagic strokes and small-vessel versus large-vessel subtypes differ in risk factors, clinical course, and recovery potential. Recognizing such constitution-based variations could therefore enhance both diagnosis and prognosis.

In addition to subtype differentiation, *Prakriti *analysis may hold prognostic value in predicting stroke recovery and long-term outcomes. Classical Ayurvedic principles suggest that individuals with *Vata*-dominant constitutions are more prone to residual deficits and slower functional recovery due to the inherent mobility and instability of *Vata*. In contrast, Kapha dominance, though associated with sluggish disease onset, may support steadier recovery once balance is restored, whereas *Pitta *influence could manifest as rapid changes, both deterioration and improvement, depending on the clinical course. These insights parallel modern findings that genetic, metabolic, and inflammatory profiles influence post-stroke neuroplasticity, rehabilitation potential, and risk of recurrence. Thus, integrating *Prakriti *profiling with conventional prognostic indicators may provide a more nuanced understanding of individual recovery trajectories, enabling personalized rehabilitation plans and improving long-term quality of life in stroke survivors.

From a therapeutic standpoint, Ayurveda’s principle of *Prakriti*-*anusara* *cikitsa* (constitution-based treatment) bears strong parallels with the modern concept of precision medicine. Just as pharmacogenomics and biomarker-driven models are increasingly applied to predict stroke outcomes and optimize drug response, *Prakriti *profiling offers a holistic, time-tested approach to individualizing management. It enables tailored interventions ranging from Panchakarma (detoxification) procedures to dietary and lifestyle modifications and drug selection, based on an individual’s constitution and disease expression. Integrating *Prakriti *insights with contemporary biomedical tools could therefore enrich predictive models, refine subtype differentiation, and advance truly personalized and integrative stroke care.

A few limitations warrant consideration. First, this review was restricted to freely accessible articles, and subscription-based studies may have provided additional insights; thus, the evidence base may be narrower than desired. Second, the subjective nature of *Prakriti *assessment introduces variability, as diagnostic interpretation often depends on the assessor’s training and methodology. This limits reproducibility and comparability across studies. Addressing this issue through the development of a validated, standardized, and objective *Prakriti *assessment tool is a critical step toward integrating Ayurvedic constitution-based prediction into mainstream stroke research.

The present review reinforces the Ayurvedic understanding of *Vata *predominance in stroke while highlighting the potential of *Prakriti *analysis to contribute to predictive, preventive, and personalized medicine. Future research should focus on refining assessment methods, conducting large-scale prospective studies, and integrating *Prakriti*-based risk profiling with conventional biomedical markers to develop robust, culturally relevant predictive models for stroke in the Indian population and beyond.

## Conclusions

The integration of *Prakriti*-based profiling into predictive medicine holds considerable promise. Unlike conventional risk scores, which are often derived from population averages and may overlook inter-individual and ethnic variations, *Prakriti *analysis provides a personalized lens by examining an individual’s physical, physiological, and psychological constitution. Such an approach could refine disease risk prediction and guide tailored lifestyle modifications, preventive strategies, and constitution-specific therapeutic interventions. Only a limited number (27) of studies were ultimately included to assess the relationship between *Prakriti *and stroke. Such a small evidence base may not provide sufficiently robust or generalizable conclusions.

For future research, we recommend conducting prospective cohort studies to evaluate the predictive validity of *Prakriti *in stroke, developing standardized and validated *Prakriti *assessment tools to ensure reproducibility, and integrating *Prakriti *profiling with biomedical and genetic markers to create robust, multidimensional risk models. Additionally, studies exploring the role of lifestyle interventions tailored to specific *Prakriti *types could provide practical strategies for stroke prevention. Ultimately, bridging Ayurvedic insights with modern biomedical research may enable a comprehensive, person-centered predictive model for stroke and other non-communicable diseases, enhancing preventive healthcare both in India and globally.
